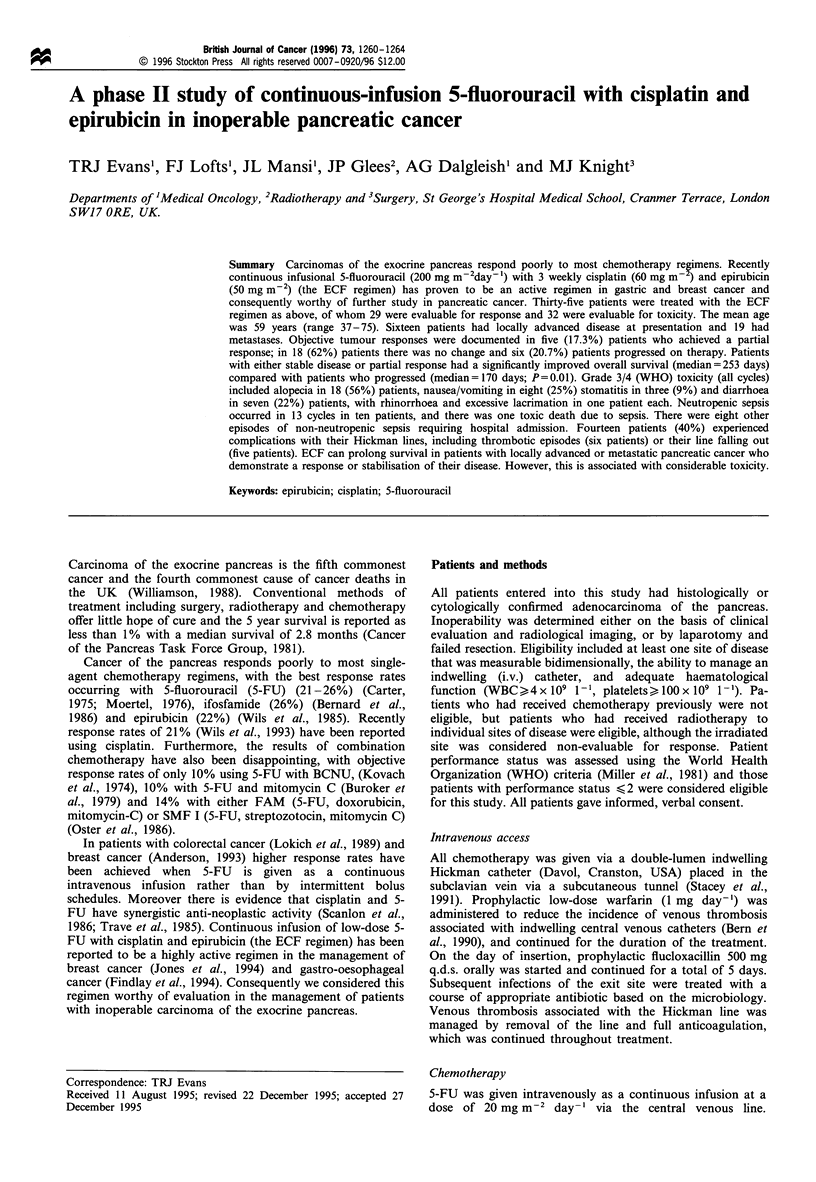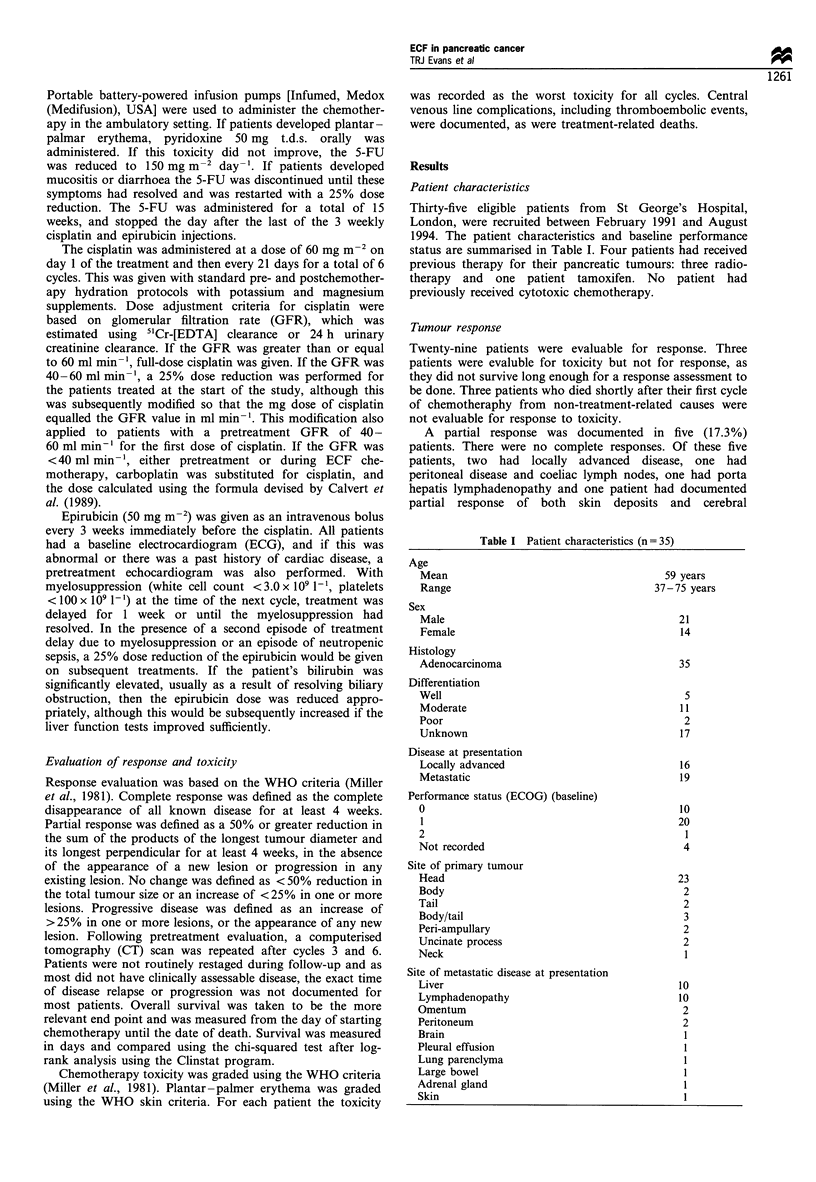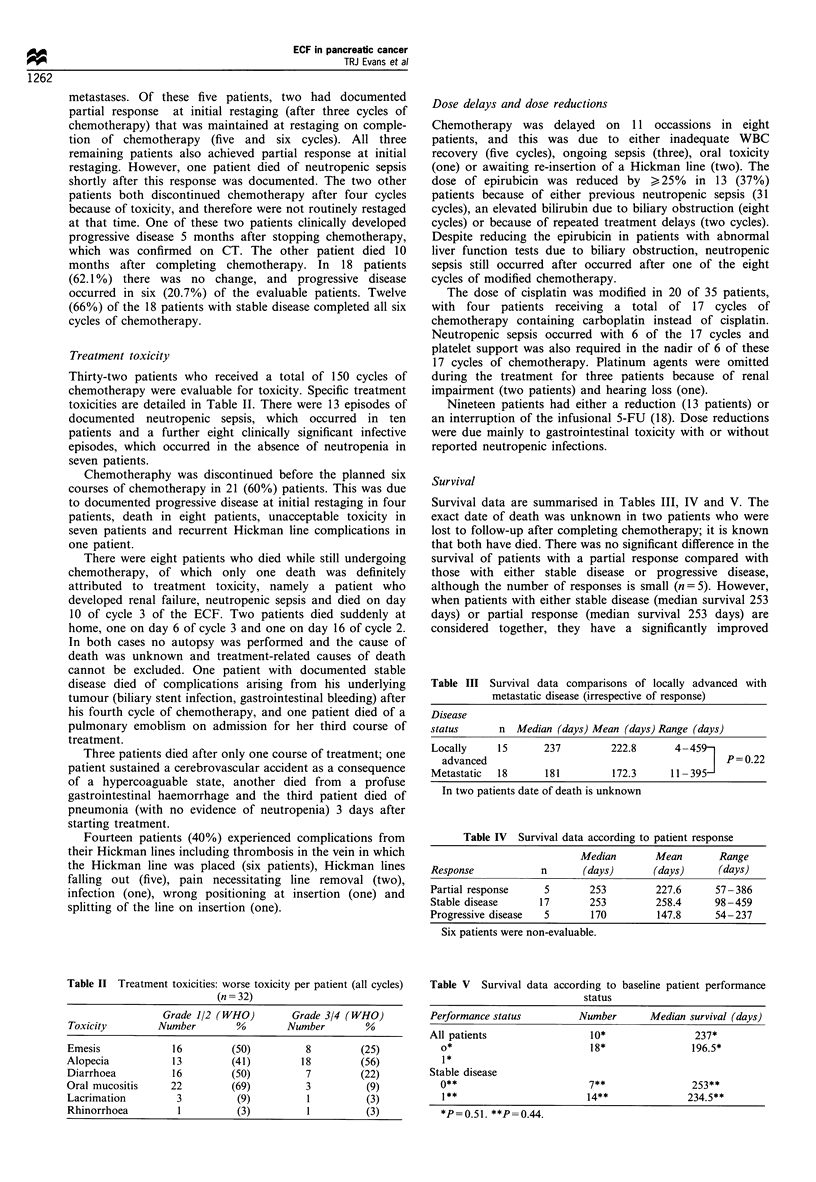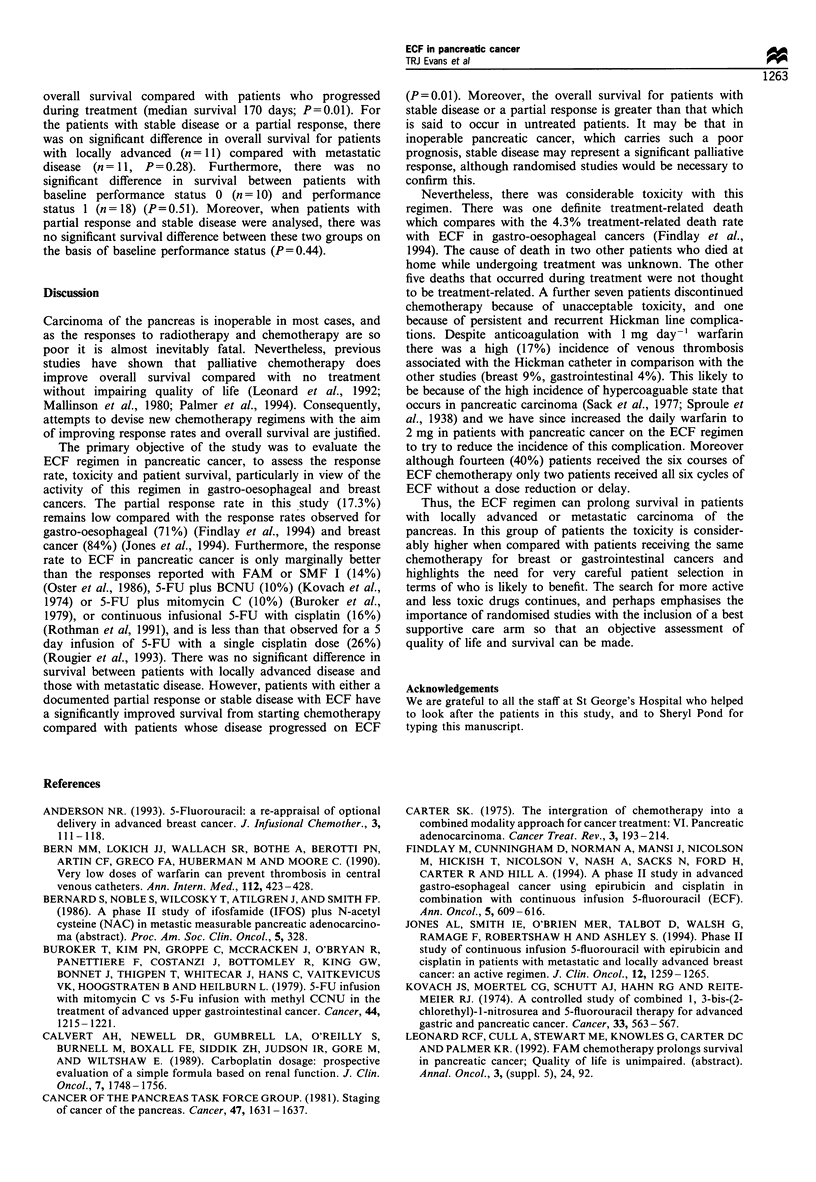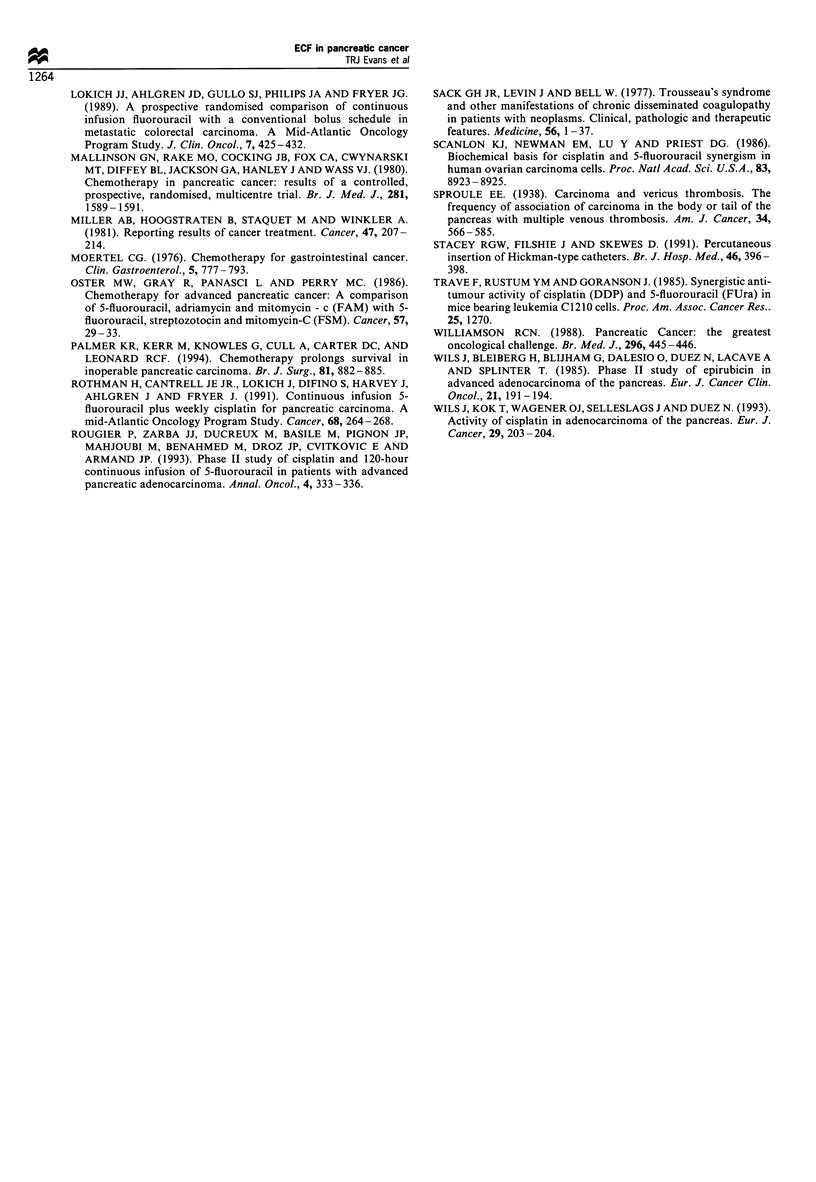# A phase II study of continuous-infusion 5-fluorouracil with cisplatin and epirubicin in inoperable pancreatic cancer.

**DOI:** 10.1038/bjc.1996.241

**Published:** 1996-05

**Authors:** T. R. Evans, F. J. Lofts, J. L. Mansi, J. P. Glees, A. G. Dalgleish, M. J. Knight

**Affiliations:** Department of Medical Oncology, St George's Hospital Medical School, London, UK.

## Abstract

Carcinomas of the exocrine pancreas respond poorly to most chemotherapy regimens. Recently continuous infusional 5-fluorouracil (200 mg m-(2)day-1) with 3 weekly cisplatin (60 mg m-2) and epirubicin (50 mg m-2) (the ECF regimen) has proven to be an active regimen in gastric and breast cancer and consequently worthy of further study in pancreatic cancer. Thirty-five patients were treated with the ECF regimen as above, of whom 29 were evaluable for response and 32 were evaluable for toxicity. The mean age was 59 years (range 37-75). Sixteen patients had locally advanced disease at presentation and 19 had metastases. Objective tumour responses were documented in five (17.3%) patients who achieved a partial response; in 18 (62%) patients there were no change and six (20.7%) patients progressed on therapy. Patients with either stable disease or partial response had a significantly improved overall survival (median = 253 days) compared with patients who progressed (median = 170 days; P = 0.01). Grade 3/4 (WHO) toxicity (all cycles) included alopecia in 18 (56%) patients, nausea/vomiting in eight (25%) stomatitis in three (9%) and diarrhoea in seven (22%) patients, with rhinorrhoea and excessive lacrimation in one patient each. Neutropenic sepsis occurred in 13 cycles in ten patients, and there was one toxic death due to sepsis. There were eight other episodes of non-neutropenic sepsis requiring hospital admission. Fourteen patients (40%) experienced complications with their Hickman lines, including thrombotic episodes (six patients) or their line falling out (five patients). ECF can prolong survival in patients with locally advanced or metastatic pancreatic cancer who demonstrate a response or stabilisation of their disease. However, this is associated with considerable toxicity.


British Journal of Cancer (1996) 73, 1260-1264
© 1996 Stockton Press All rights reserved 0007-0920/96 $12.00

### A phase II study of continuous-infusion 5-fluorouracil with cisplatin and epirubicin in inoperable pancreatic cancer

TRJ Evans[1], FJ Lofts[1], JL Mansi[1], JP Glees[2], AG Dalgleish[1] and MJ Knight[3]

Departments of [1]Medical Oncology, [2]Radiotherapy and [3]Surgery, St George's Hospital Medical School, Cranmer Terrace, London SW17 0RE, UK.

**Summary** Carcinomas of the exocrine pancreas respond poorly to most chemotherapy regimens. Recently continuous infusional 5-fluorouracil (200 mg $m^{-2}day^{-1}$) with 3 weekly cisplatin (60 mg $m^{-2}$) and epirubicin (50 mg $m^{-2}$) (the ECF regimen) has proven to be an active regimen in gastric and breast cancer and consequently worthy of further study in pancreatic cancer. Thirty-five patients were treated with the ECF regimen as above, of whom 29 were evaluable for response and 32 were evaluable for toxicity. The mean age was 59 years (range 37-75). Sixteen patients had locally advanced disease at presentation and 19 had metastases. Objective tumour responses were documented in five (17.3%) patients who achieved a partial response; in 18 (62%) patients there was no change and six (20.7%) patients progressed on therapy. Patients with either stable disease or partial response had a significantly improved overall survival (median = 253 days) compared with patients who progressed (median = 170 days; $P$ = 0.01). Grade 3/4 (WHO) toxicity (all cycles) included alopecia in 18 (56%) patients, nausea/vomiting in eight (25%) stomatitis in three (9%) and diarrhoea in seven (22%) patients, with rhinorrhoea and excessive lacrimation in one patient each. Neutropenic sepsis occurred in 13 cycles in ten patients, and there was one toxic death due to sepsis. There were eight other episodes of non-neutropenic sepsis requiring hospital admission. Fourteen patients (40%) experienced complications with their Hickman lines, including thrombotic episodes (six patients) or their line falling out (five patients). ECF can prolong survival in patients with locally advanced or metastatic pancreatic cancer who demonstrate a response or stabilisation of their disease. However, this is associated with considerable toxicity.

**Keywords:** epirubicin; cisplatin; 5-fluorouracil

Carcinoma of the exocrine pancreas is the fifth commonest cancer and the fourth commonest cause of cancer deaths in the UK (Williamson, 1988). Conventional methods of treatment including surgery, radiotherapy and chemotherapy offer little hope of cure and the 5 year survival is reported as less than 1% with a median survival of 2.8 months (Cancer of the Pancreas Task Force Group, 1981).

Cancer of the pancreas responds poorly to most single-agent chemotherapy regimens, with the best response rates occurring with 5-fluorouracil (5-FU) (21-26%) (Carter, 1975; Moertel, 1976), ifosfamide (26%) (Bernard *et al.*, 1986) and epirubicin (22%) (Wils *et al.*, 1985). Recently response rates of 21% (Wils *et al.*, 1993) have been reported using cisplatin. Furthermore, the results of combination chemotherapy have also been disappointing, with objective response rates of only 10% using 5-FU with BCNU, (Kovach *et al.*, 1974), 10% with 5-FU and mitomycin C (Buroker *et al.*, 1979) and 14% with either FAM (5-FU, doxorubicin, mitomycin-C) or SMF I (5-FU, streptozotocin, mitomycin C) (Oster *et al.*, 1986).

In patients with colorectal cancer (Lokich *et al.*, 1989) and breast cancer (Anderson, 1993) higher response rates have been achieved when 5-FU is given as a continuous intravenous infusion rather than by intermittent bolus schedules. Moreover there is evidence that cisplatin and 5-FU have synergistic anti-neoplastic activity (Scanlon *et al.*, 1986; Trave *et al.*, 1985). Continuous infusion of low-dose 5-FU with cisplatin and epirubicin (the ECF regimen) has been reported to be a highly active regimen in the management of breast cancer (Jones *et al.*, 1994) and gastro-oesophageal cancer (Findlay *et al.*, 1994). Consequently we considered this regimen worthy of evaluation in the management of patients with inoperable carcinoma of the exocrine pancreas.

Correspondence: TRJ Evans
Received 11 August 1995; revised 22 December 1995; accepted 27 December 1995

### Patients and methods

All patients entered into this study had histologically or cytologically confirmed adenocarcinoma of the pancreas. Inoperability was determined either on the basis of clinical evaluation and radiological imaging, or by laparotomy and failed resection. Eligibility included at least one site of disease that was measurable bidimensionally, the ability to manage an indwelling (i.v.) catheter, and adequate haematological function (WBC $\geqslant 4 \times 10^9$ $l^{-1}$, platelets $\geqslant 100 \times 10^9$ $l^{-1}$). Patients who had received chemotherapy previously were not eligible, but patients who had received radiotherapy to individual sites of disease were eligible, although the irradiated site was considered non-evaluable for response. Patient performance status was assessed using the World Health Organization (WHO) criteria (Miller *et al.*, 1981) and those patients with performance status $\leqslant 2$ were considered eligible for this study. All patients gave informed, verbal consent.

### *Intravenous access*

All chemotherapy was given via a double-lumen indwelling Hickman catheter (Davol, Cranston, USA) placed in the subclavian vein via a subcutaneous tunnel (Stacey *et al.*, 1991). Prophylactic low-dose warfarin (1 mg $day^{-1}$) was administered to reduce the incidence of venous thrombosis associated with indwelling central venous catheters (Bern *et al.*, 1990), and continued for the duration of the treatment. On the day of insertion, prophylactic flucloxacillin 500 mg q.d.s. orally was started and continued for a total of 5 days. Subsequent infections of the exit site were treated with a course of appropriate antibiotic based on the microbiology. Venous thrombosis associated with the Hickman line was managed by removal of the line and full anticoagulation, which was continued throughout treatment.

### *Chemotherapy*

5-FU was given intravenously as a continuous infusion at a dose of 20 mg $m^{-2}$ $day^{-1}$ via the central venous line.

Portable battery-powered infusion pumps [Infumed, Medox (Medifusion), USA] were used to administer the chemotherapy in the ambulatory setting. If patients developed plantar–palmar erythema, pyridoxine 50 mg t.d.s. orally was administered. If this toxicity did not improve, the 5-FU was reduced to 150 mg $m^{-2}$ $day^{-1}$. If patients developed mucositis or diarrhoea the 5-FU was discontinued until these symptoms had resolved and was restarted with a 25% dose reduction. The 5-FU was administered for a total of 15 weeks, and stopped the day after the last of the 3 weekly cisplatin and epirubicin injections.

The cisplatin was administered at a dose of 60 mg $m^{-2}$ on day 1 of the treatment and then every 21 days for a total of 6 cycles. This was given with standard pre- and postchemotherapy hydration protocols with potassium and magnesium supplements. Dose adjustment criteria for cisplatin were based on glomerular filtration rate (GFR), which was estimated using $^{51}$Cr-[EDTA] clearance or 24 h urinary creatinine clearance. If the GFR was greater than or equal to 60 ml $min^{-1}$, full-dose cisplatin was given. If the GFR was 40–60 ml $min^{-1}$, a 25% dose reduction was performed for the patients treated at the start of the study, although this was subsequently modified so that the mg dose of cisplatin equalled the GFR value in ml $min^{-1}$. This modification also applied to patients with a pretreatment GFR of 40–60 ml $min^{-1}$ for the first dose of cisplatin. If the GFR was $<40$ ml $min^{-1}$, either pretreatment or during ECF chemotherapy, carboplatin was substituted for cisplatin, and the dose calculated using the formula devised by Calvert *et al.* (1989).

Epirubicin (50 mg $m^{-2}$) was given as an intravenous bolus every 3 weeks immediately before the cisplatin. All patients had a baseline electrocardiogram (ECG), and if this was abnormal or there was a past history of cardiac disease, a pretreatment echocardiogram was also performed. With myelosuppression (white cell count $<3.0 \times 10^9$ $l^{-1}$, platelets $<100 \times 10^9$ $l^{-1}$) at the time of the next cycle, treatment was delayed for 1 week or until the myelosuppression had resolved. In the presence of a second episode of treatment delay due to myelosuppression or an episode of neutropenic sepsis, a 25% dose reduction of the epirubicin would be given on subsequent treatments. If the patient's bilirubin was significantly elevated, usually as a result of resolving biliary obstruction, then the epirubicin dose was reduced appropriately, although this would be subsequently increased if the liver function tests improved sufficiently.

### *Evaluation of response and toxicity*

Response evaluation was based on the WHO criteria (Miller *et al.*, 1981). Complete response was defined as the complete disappearance of all known disease for at least 4 weeks. Partial response was defined as a 50% or greater reduction in the sum of the products of the longest tumour diameter and its longest perpendicular for at least 4 weeks, in the absence of the appearance of a new lesion or progression in any existing lesion. No change was defined as <50% reduction in the total tumour size or an increase of <25% in one or more lesions. Progressive disease was defined as an increase of >25% in one or more lesions, or the appearance of any new lesion. Following pretreatment evaluation, a computerised tomography (CT) scan was repeated after cycles 3 and 6. Patients were not routinely restaged during follow-up and as most did not have clinically assessable disease, the exact time of disease relapse or progression was not documented for most patients. Overall survival was taken to be the more relevant end point and was measured from the day of starting chemotherapy until the date of death. Survival was measured in days and compared using the chi-squared test after log-rank analysis using the Clinstat program.

Chemotherapy toxicity was graded using the WHO criteria (Miller *et al.*, 1981). Plantar–palmer erythema was graded using the WHO skin criteria. For each patient the toxicity was recorded as the worst toxicity for all cycles. Central venous line complications, including thromboembolic events, were documented, as were treatment-related deaths.

### Results

### *Patient characteristics*

Thirty-five eligible patients from St George's Hospital, London, were recruited between February 1991 and August 1994. The patient characteristics and baseline performance status are summarised in Table I. Four patients had received previous therapy for their pancreatic tumours: three radiotherapy and one patient tamoxifen. No patient had previously received cytotoxic chemotherapy.

### *Tumour response*

Twenty-nine patients were evaluable for response. Three patients were evaluble for toxicity but not for response, as they did not survive long enough for a response assessment to be done. Three patients who died shortly after their first cycle of chemotheraphy from non-treatment-related causes were not evaluable for response to toxicity.

A partial response was documented in five (17.3%) patients. There were no complete responses. Of these five patients, two had locally advanced disease, one had peritoneal disease and coeliac lymph nodes, one had porta hepatis lymphadenopathy and one patient had documented partial response of both skin deposits and cerebral

**Table I** Patient characteristics (n = 35)

| | |
|---|---|
| Age | |
| Mean | 59 years |
| Range | 37–75 years |
| Sex | |
| Male | 21 |
| Female | 14 |
| Histology | |
| Adenocarcinoma | 35 |
| Differentiation | |
| Well | 5 |
| Moderate | 11 |
| Poor | 2 |
| Unknown | 17 |
| Disease at presentation | |
| Locally advanced | 16 |
| Metastatic | 19 |
| Performance status (ECOG) (baseline) | |
| 0 | 10 |
| 1 | 20 |
| 2 | 1 |
| Not recorded | 4 |
| Site of primary tumour | |
| Head | 23 |
| Body | 2 |
| Tail | 2 |
| Body/tail | 3 |
| Peri-ampullary | 2 |
| Uncinate process | 2 |
| Neck | 1 |
| Site of metastatic disease at presentation | |
| Liver | 10 |
| Lymphadenopathy | 10 |
| Omentum | 2 |
| Peritoneum | 2 |
| Brain | 1 |
| Pleural effusion | 1 |
| Lung parenclyma | 1 |
| Large bowel | 1 |
| Adrenal gland | 1 |
| Skin | 1 |

metastases. Of these five patients, two had documented partial response at initial restaging (after three cycles of chemotherapy) that was maintained at restaging on completion of chemotherapy (five and six cycles). All three remaining patients also achieved partial response at initial restaging. However, one patient died of neutropenic sepsis shortly after this response was documented. The two other patients both discontinued chemotherapy after four cycles because of toxicity, and therefore were not routinely restaged at that time. One of these two patients clinically developed progressive disease 5 months after stopping chemotherapy, which was confirmed on CT. The other patient died 10 months after completing chemotherapy. In 18 patients (62.1%) there was no change, and progressive disease occurred in six (20.7%) of the evaluable patients. Twelve (66%) of the 18 patients with stable disease completed all six cycles of chemotherapy.

*Treatment toxicity*

Thirty-two patients who received a total of 150 cycles of chemotherapy were evaluable for toxicity. Specific treatment toxicities are detailed in Table II. There were 13 episodes of documented neutropenic sepsis, which occurred in ten patients and a further eight clinically significant infective episodes, which occurred in the absence of neutropenia in seven patients.

Chemotheraphy was discontinued before the planned six courses of chemotherapy in 21 (60%) patients. This was due to documented progressive disease at initial restaging in four patients, death in eight patients, unacceptable toxicity in seven patients and recurrent Hickman line complications in one patient.

There were eight patients who died while still undergoing chemotherapy, of which only one death was definitely attributed to treatment toxicity, namely a patient who developed renal failure, neutropenic sepsis and died on day 10 of cycle 3 of the ECF. Two patients died suddenly at home, one on day 6 of cycle 3 and one on day 16 of cycle 2. In both cases no autopsy was performed and the cause of death was unknown and treatment-related causes of death cannot be excluded. One patient with documented stable disease died of complications arising from his underlying tumour (biliary stent infection, gastrointestinal bleeding) after his fourth cycle of chemotherapy, and one patient died of a pulmonary emoblism on admission for her third course of treatment.

Three patients died after only one course of treatment; one patient sustained a cerebrovascular accident as a consequence of a hypercoaguable state, another died from a profuse gastrointestinal haemorrhage and the third patient died of pneumonia (with no evidence of neutropenia) 3 days after starting treatment.

Fourteen patients (40%) experienced complications from their Hickman lines including thrombosis in the vein in which the Hickman line was placed (six patients), Hickman lines falling out (five), pain necessitating line removal (two), infection (one), wrong positioning at insertion (one) and splitting of the line on insertion (one).

**Table II** Treatment toxicities: worse toxicity per patient (all cycles) ($n=32$)

| Toxicity | Grade 1/2 (WHO) Number | % | Grade 3/4 (WHO) Number | % |
|---|---|---|---|---|
| Emesis | 16 | (50) | 8 | (25) |
| Alopecia | 13 | (41) | 18 | (56) |
| Diarrhoea | 16 | (50) | 7 | (22) |
| Oral mucositis | 22 | (69) | 3 | (9) |
| Lacrimation | 3 | (9) | 1 | (3) |
| Rhinorrhoea | 1 | (3) | 1 | (3) |

*Dose delays and dose reductions*

Chemotherapy was delayed on 11 occassions in eight patients, and this was due to either inadequate WBC recovery (five cycles), ongoing sepsis (three), oral toxicity (one) or awaiting re-insertion of a Hickman line (two). The dose of epirubicin was reduced by $\geqslant$25% in 13 (37%) patients because of either previous neutropenic sepsis (31 cycles), an elevated bilirubin due to biliary obstruction (eight cycles) or because of repeated treatment delays (two cycles). Despite reducing the epirubicin in patients with abnormal liver function tests due to biliary obstruction, neutropenic sepsis still occurred after occurred after one of the eight cycles of modified chemotherapy.

The dose of cisplatin was modified in 20 of 35 patients, with four patients receiving a total of 17 cycles of chemotherapy containing carboplatin instead of cisplatin. Neutropenic sepsis occurred with 6 of the 17 cycles and platelet support was also required in the nadir of 6 of these 17 cycles of chemotherapy. Platinum agents were omitted during the treatment for three patients because of renal impairment (two patients) and hearing loss (one).

Nineteen patients had either a reduction (13 patients) or an interruption of the infusional 5-FU (18). Dose reductions were due mainly to gastrointestinal toxicity with or without reported neutropenic infections.

*Survival*

Survival data are summarised in Tables III, IV and V. The exact date of death was unknown in two patients who were lost to follow-up after completing chemotherapy; it is known that both have died. There was no significant difference in the survival of patients with a partial response compared with those with either stable disease or progressive disease, although the number of responses is small ($n=5$). However, when patients with either stable disease (median survival 253 days) or partial response (median survival 253 days) are considered together, they have a significantly improved

**Table III** Survival data comparisons of locally advanced with metastatic disease (irrespective of response)

| Disease status | n | Median (days) | Mean (days) | Range (days) | |
|---|---|---|---|---|---|
| Locally advanced | 15 | 237 | 222.8 | 4–459 | $P=0.22$ |
| Metastatic | 18 | 181 | 172.3 | 11–395 | |

In two patients date of death is unknown

**Table IV** Survival data according to patient response

| Response | n | Median (days) | Mean (days) | Range (days) |
|---|---|---|---|---|
| Partial response | 5 | 253 | 227.6 | 57–386 |
| Stable disease | 17 | 253 | 258.4 | 98–459 |
| Progressive disease | 5 | 170 | 147.8 | 54–237 |

Six patients were non-evaluable.

**Table V** Survival data according to baseline patient performance status

| Performance status | Number | Median survival (days) |
|---|---|---|
| All patients | 10* | 237* |
| o* | 18* | 196.5* |
| 1* | | |
| Stable disease | | |
| 0** | 7** | 253** |
| 1** | 14** | 234.5** |

*$P=0.51$. **$P=0.44$.

overall survival compared with patients who progressed during treatment (median survival 170 days; $P=0.01$). For the patients with stable disease or a partial response, there was on significant difference in overall survival for patients with locally advanced ($n=11$) compared with metastatic disease ($n=11$, $P=0.28$). Furthermore, there was no significant difference in survival between patients with baseline performance status 0 ($n=10$) and performance status 1 ($n=18$) ($P=0.51$). Moreover, when patients with partial response and stable disease were analysed, there was no significant survival difference between these two groups on the basis of baseline performance status ($P=0.44$).

### Discussion

Carcinoma of the pancreas is inoperable in most cases, and as the responses to radiotherapy and chemotherapy are so poor it is almost inevitably fatal. Nevertheless, previous studies have shown that palliative chemotherapy does improve overall survival compared with no treatment without impairing quality of life (Leonard *et al.*, 1992; Mallinson *et al.*, 1980; Palmer *et al.*, 1994). Consequently, attempts to devise new chemotherapy regimens with the aim of improving response rates and overall survival are justified.

The primary objective of the study was to evaluate the ECF regimen in pancreatic cancer, to assess the response rate, toxicity and patient survival, particularly in view of the activity of this regimen in gastro-oesophageal and breast cancers. The partial response rate in this study (17.3%) remains low compared with the response rates observed for gastro-oesophageal (71%) (Findlay *et al.*, 1994) and breast cancer (84%) (Jones *et al.*, 1994). Furthermore, the response rate to ECF in pancreatic cancer is only marginally better than the responses reported with FAM or SMF I (14%) (Oster *et al.*, 1986), 5-FU plus BCNU (10%) (Kovach *et al.*, 1974) or 5-FU plus mitomycin C (10%) (Buroker *et al.*, 1979), or continuous infusional 5-FU with cisplatin (16%) (Rothman *et al*, 1991), and is less than that observed for a 5 day infusion of 5-FU with a single cisplatin dose (26%) (Rougier *et al.*, 1993). There was no significant difference in survival between patients with locally advanced disease and those with metastatic disease. However, patients with either a documented partial response or stable disease with ECF have a significantly improved survival from starting chemotherapy compared with patients whose disease progressed on ECF ($P=0.01$). Moreover, the overall survival for patients with stable disease or a partial response is greater than that which is said to occur in untreated patients. It may be that in inoperable pancreatic cancer, which carries such a poor prognosis, stable disease may represent a significant palliative response, although randomised studies would be necessary to confirm this.

Nevertheless, there was considerable toxicity with this regimen. There was one definite treatment-related death which compares with the 4.3% treatment-related death rate with ECF in gastro-oesophageal cancers (Findlay *et al.*, 1994). The cause of death in two other patients who died at home while undergoing treatment was unknown. The other five deaths that occurred during treatment were not thought to be treatment-related. A further seven patients discontinued chemotherapy because of unacceptable toxicity, and one because of persistent and recurrent Hickman line complications. Despite anticoagulation with 1 mg $\text{day}^{-1}$ warfarin there was a high (17%) incidence of venous thrombosis associated with the Hickman catheter in comparison with the other studies (breast 9%, gastrointestinal 4%). This likely to be because of the high incidence of hypercoaguable state that occurs in pancreatic carcinoma (Sack *et al.*, 1977; Sproule *et al.*, 1938) and we have since increased the daily warfarin to 2 mg in patients with pancreatic cancer on the ECF regimen to try to reduce the incidence of this complication. Moreover although fourteen (40%) patients received the six courses of ECF chemotherapy only two patients received all six cycles of ECF without a dose reduction or delay.

Thus, the ECF regimen can prolong survival in patients with locally advanced or metastatic carcinoma of the pancreas. In this group of patients the toxicity is considerably higher when compared with patients receiving the same chemotherapy for breast or gastrointestinal cancers and highlights the need for very careful patient selection in terms of who is likely to benefit. The search for more active and less toxic drugs continues, and perhaps emphasises the importance of randomised studies with the inclusion of a best supportive care arm so that an objective assessment of quality of life and survival can be made.

**Acknowledgements**
We are grateful to all the staff at St George's Hospital who helped to look after the patients in this study, and to Sheryl Pond for typing this manuscript.

### References

ANDERSON NR. (1993). 5-Fluorouracil: a re-appraisal of optional delivery in advanced breast cancer. *J. Infusional Chemother.*, **3**, 111–118.

BERN MM, LOKICH JJ, WALLACH SR, BOTHE A, BEROTTI PN, ARTIN CF, GRECO FA, HUBERMAN M AND MOORE C. (1990). Very low doses of warfarin can prevent thrombosis in central venous catheters. *Ann. Intern. Med.*, **112**, 423–428.

BERNARD S, NOBLE S, WILCOSKY T, ATILGREN J, AND SMITH FP. (1986). A phase II study of ifosfamide (IFOS) plus N-acetyl cysteine (NAC) in metastic measurable pancreatic adenocarcinoma (abstract). *Proc. Am. Soc. Clin. Oncol.*, **5**, 328.

BUROKER T, KIM PN, GROPPE C, MCCRACKEN J, O'BRYAN R, PANETTIERE F, COSTANZI J, BOTTOMLEY R, KING GW, BONNET J, THIGPEN T, WHITECAR J, HANS C, VAITKEVICUS VK, HOOGSTRATEN B AND HEILBURN L. (1979). 5-FU infusion with mitomycin C vs 5-Fu infusion with methyl CCNU in the treatment of advanced upper gastrointestinal cancer. *Cancer*, **44**, 1215–1221.

CALVERT AH, NEWELL DR, GUMBRELL LA, O'REILLY S, BURNELL M, BOXALL FE, SIDDIK ZH, JUDSON IR, GORE M, AND WILTSHAW E. (1989). Carboplatin dosage: prospective evaluation of a simple formula based on renal function. *J. Clin. Oncol.*, **7**, 1748–1756.

CANCER OF THE PANCREAS TASK FORCE GROUP. (1981). Staging of cancer of the pancreas. *Cancer*, **47**, 1631–1637.

CARTER SK. (1975). The intergration of chemotherapy into a combined modality approach for cancer treatment: VI. Pancreatic adenocarcinoma. *Cancer Treat. Rev.*, **3**, 193–214.

FINDLAY M, CUNNINGHAM D, NORMAN A, MANSI J, NICOLSON M, HICKISH T, NICOLSON V, NASH A, SACKS N, FORD H, CARTER R AND HILL A. (1994). A phase II study in advanced gastro-esophageal cancer using epirubicin and cisplatin in combination with continuous infusion 5-fluorouracil (ECF). *Ann. Oncol.*, **5**, 609–616.

JONES AL, SMITH IE, O'BRIEN MER, TALBOT D, WALSH G, RAMAGE F, ROBERTSHAW H AND ASHLEY S. (1994). Phase II study of continuous infusion 5-fluorouracil with epirubicin and cisplatin in patients with metastatic and locally advanced breast cancer: an active regimen. *J. Clin. Oncol.*, **12**, 1259–1265.

KOVACH JS, MOERTEL CG, SCHUTT AJ, HAHN RG AND REITEMEIER RJ. (1974). A controlled study of combined 1, 3-bis-(2-chlorethyl)-1-nitrosurea and 5-fluorouracil therapy for advanced gastric and pancreatic cancer. *Cancer*, **33**, 563–567.

LEONARD RCF, CULL A, STEWART ME, KNOWLES G, CARTER DC AND PALMER KR. (1992). FAM chemotherapy prolongs survival in pancreatic cancer; Quality of life is unimpaired. (abstract). *Annal. Oncol.*, **3**, (suppl. 5), 24, 92.

LOKICH JJ, AHLGREN JD, GULLO SJ, PHILIPS JA AND FRYER JG. (1989). A prospective randomised comparison of continuous infusion fluorouracil with a conventional bolus schedule in metastatic colorectal carcinoma. A Mid-Atlantic Oncology Program Study. *J. Clin. Oncol.*, **7**, 425–432.

MALLINSON GN, RAKE MO, COCKING JB, FOX CA, CWYNARSKI MT, DIFFEY BL, JACKSON GA, HANLEY J AND WASS VJ. (1980). Chemotherapy in pancreatic cancer: results of a controlled, prospective, randomised, multicentre trial. *Br. J. Med. J.*, **281**, 1589–1591.

MILLER AB, HOOGSTRATEN B, STAQUET M AND WINKLER A. (1981). Reporting results of cancer treatment. *Cancer*, **47**, 207–214.

MOERTEL CG. (1976). Chemotherapy for gastrointestinal cancer. *Clin. Gastroenterol.*, **5**, 777–793.

OSTER MW, GRAY R, PANASCI L AND PERRY MC. (1986). Chemotherapy for advanced pancreatic cancer: A comparison of 5-fluorouracil, adriamycin and mitomycin - c (FAM) with 5-fluorouracil, streptozotocin and mitomycin-C (FSM). *Cancer*, **57**, 29–33.

PALMER KR, KERR M, KNOWLES G, CULL A, CARTER DC, AND LEONARD RCF. (1994). Chemotherapy prolongs survival in inoperable pancreatic carcinoma. *Br. J. Surg.*, **81**, 882–885.

ROTHMAN H, CANTRELL JE JR., LOKICH J, DIFINO S, HARVEY J, AHLGREN J AND FRYER J. (1991). Continuous infusion 5-fluorouracil plus weekly cisplatin for pancreatic carcinoma. A mid-Atlantic Oncology Program Study. *Cancer*, **68**, 264–268.

ROUGIER P, ZARBA JJ, DUCREUX M, BASILE M, PIGNON JP, MAHJOUBI M, BENAHMED M, DROZ JP, CVITKOVIC E AND ARMAND JP. (1993). Phase II study of cisplatin and 120-hour continuous infusion of 5-fluorouracil in patients with advanced pancreatic adenocarcinoma. *Annal. Oncol.*, **4**, 333–336.

SACK GH JR, LEVIN J AND BELL W. (1977). Trousseau's syndrome and other manifestations of chronic disseminated coagulopathy in patients with neoplasms. Clinical, pathologic and therapeutic features. *Medicine*, **56**, 1–37.

SCANLON KJ, NEWMAN EM, LU Y AND PRIEST DG. (1986). Biochemical basis for cisplatin and 5-fluorouracil synergism in human ovarian carcinoma cells. *Proc. Natl Acad. Sci. U.S.A.*, **83**, 8923–8925.

SPROULE EE. (1938). Carcinoma and vericus thrombosis. The frequency of association of carcinoma in the body or tail of the pancreas with multiple venous thrombosis. *Am. J. Cancer*, **34**, 566–585.

STACEY RGW, FILSHIE J AND SKEWES D. (1991). Percutaneous insertion of Hickman-type catheters. *Br. J. Hosp. Med.*, **46**, 396–398.

TRAVE F, RUSTUM YM AND GORANSON J. (1985). Synergistic anti-tumour activity of cisplatin (DDP) and 5-fluorouracil (FUra) in mice bearing leukemia C1210 cells. *Proc. Am. Assoc. Cancer Res.*. **25**, 1270.

WILLIAMSON RCN. (1988). Pancreatic Cancer: the greatest oncological challenge. *Br. Med. J.*, **296**, 445–446.

WILS J, BLEIBERG H, BLIJHAM G, DALESIO O, DUEZ N, LACAVE A AND SPLINTER T. (1985). Phase II study of epirubicin in advanced adenocarcinoma of the pancreas. *Eur. J. Cancer Clin. Oncol.*, **21**, 191–194.

WILS J, KOK T, WAGENER OJ, SELLESLAGS J AND DUEZ N. (1993). Activity of cisplatin in adenocarcinoma of the pancreas. *Eur. J. Cancer*, **29**, 203–204.